# A genome-wide association and polygenic risk score study on abnormal electrocardiogram in a Chinese population

**DOI:** 10.1038/s41598-021-84135-7

**Published:** 2021-02-25

**Authors:** Mengqiao Wang, Jiaqi Gao, Yang Shi, Xing Zhao

**Affiliations:** 1 Department of Epidemiology and Biostatistics, West China School of Public Health and West China Fourth Hospital, Sichuan University, Renmin South Road 16, Chengdu, 610041 Sichuan People’s Republic of China; 2 Department of Population Health Science, Medical College of Georgia, Augusta University, 1120 15th Street, Augusta, GA 30912 USA

**Keywords:** Genetic association study, Predictive markers

## Abstract

Electrocardiography is a common and widely-performed medical examination based on the measurement and evaluation of electrocardiogram (ECG) to assess the up-to-date cardiac rhythms and thus suggest the health conditions of cardiovascular system and on a larger level the individual’s wellness. Abnormal ECG assessment from the detection of abnormal heart rhythms may have clinical implications including blood clots in formation, ongoing heart attack, coronary artery blockage, etc. Past genetic-phenotypic research focused primarily on the physical parameters of ECG but not the medical evaluation. To unbiasedly uncover the underlying links of genetic variants with normal vs*.* abnormal ECG assessment, a genome-wide association study (GWAS) is carried out in a 1006-participant cohort of Chinese population effectively genotyped for 243487 single nucleotide polymorphisms (SNPs). Both age and sex are influential factors, and six novel SNPs are identified for potential association with abnormal ECG. With the selected SNPs, a polygenic risk score (PRS) differentiates the case–control subgroups, and correlates well with increased risk of abnormal ECG. The findings are reproduced in an independent validation cohort. The derived PRS may function as a potential biomarker for prospectively screening the high-risk subgroup of heart issues in the Chinese population.

## Introduction

Electrocardiogram (abbreviated as ECG or EKG) is a non-invasive test of the electrical activity of cardiac rhythms that may indicate normal or abnormal cardiovascular conditions including cardiac hypertrophy, arrhythmias, conduction defects etc. ECG tracings consist of three major components during a single heartbeat: the P wave (generally upwards and indicating atrial depolarization)^1^, the QRS complex (of greater amplitude and indicating ventricular depolarization)^2^, and the T wave (smaller upwards wave than the QRS and indicating ventricular repolarization)^3^. Additionally, the PR reflects the time the electrical impulse takes to travel from the sinus node through the atrioventricular node. Abnormal rhythms exist as abnormally slow or fast heart rate or an irregular cardiac rhythm. An abnormal P-wave may serve as predictors for atrial arrhythmias and atrial enlargement^4^. A premature ventricular contraction or a ventricular rhythm can be revealed as a prolonged QRS duration which is associated with mortality in both the general population and in patients with cardiac diseases^5–9^. T waves can be helpful in a variety of pathologies: as an example, tall T waves in anterior chest lead III, aVR, and V1 with a negative QRS complex may suggest acute myocardial ischemia^10^. QT interval measures myocardial depolarization and repolarization time, while shortening or prolongation of QT interval is associated with an increased risk for arrhythmias and sudden cardiac death (SCD)^11,12^. Abnormal PR interval is a risk factor for atrial fibrillation and heart block^13^. Through ECG examination and the following evaluation by certified medical staffs (e.g. assessment of rhythm, range of heart rate, measurement of tracing intervals and segments), abnormality on cardiac rhythms could be detected early. Therefore, normal or abnormal ECG assessment is a key medical and clinical evaluation that provides the doctors with instrumental evidence-based revelation on individuals’ health conditions.

Both genetic and environmental factors contribute to abnormal cardiac rhythms, and regarding the mode of inheritance for such a complex trait, it is highly likely that a collection of rather than a single genetic variant may be involved. Genome-wide association study, or GWAS, aims to genotype the genome of a population in high-throughput manner and unbiasedly screen for loci significantly associated with the phenotype-of-interest. A few pioneering GWASs for various ECG phenotypes have found over 500 risk variants^14^, validating the underlying genetic basis^15–19^. The QT interval was one of the first traits investigated at the outset of the GWAS era^20^. Heritability estimates of QT interval are between 30 and 40%^21^. Previous GWAS identified 45 loci, of which 35 have been proved in QT-IGC, containing variants in genes that modestly influence QT interval, such as *GBF1*, *AZIN1*, *CREBBP*, and *KCNQ1*^22,23^. QRS interval duration is a quantitative trait influenced by multiple genetic factors and as well by both age and gender^24,25^. In addition, the heritability of QRS duration is estimated to be 35–55% from twin and family studies^26–29^. An exome-chip meta-analysis has identified 34 single nucleotide polymorphisms (SNPs) associated with QRS duration^30^, confirming 20 out of the 29 previously identified loci, including *KLHL38*^31,32^, *DLEC1*^33^, *NACA*^34^, and *ADAMTS6*^35^. However, there were so far few GWASs with the qualitative assessment of ECG results as the outcome so it remains unclear if any genetic variants may be linked to abnormal ECG status.

Moreover, like many biometric parameters, ECG features are known to vary by ethnicity^36,37^. Most of the published GWASs for ECG traits have been carried out in populations of European descent^13,38–40^, but not in populations residing in populous regions of high incidence in cardiovascular diseases such as China. In-parallel GWASs in different ethnic groups are informative in both validating common biomarkers and discovering ethnicity-specific genetic links. To our knowledge, no large-sample GWAS on the assessment of ECG has been conducted for a cohort of Chinese population, and this study aims to fill this gap to explore the association of any known or novel SNPs with normal vs. abnormal ECG assessment in a Chinese cohort.

## Methods

### Study design and procedure

A group of adult residents living in Sichuan Province of P.R. China were recruited as a regional cohort and among them, 1006 individuals participated in the project studying the association between genomic polymorphisms and the evaluation of electrocardiogram status. For ECG measurement, individual participant was informed of the standard procedure in advance and was given a grace period of 20 min to calm down before measurement by certified medical staffs using a portable electrocardiogram machine that measures and records heart activity (setting at 1 cm/mV). A conventional 12-lead electrocardiogram (I, II, III, aVR, aVL, aVF, V1, V2, V3, V4, V5, V6) measures the overall magnitude and direction of the individuals’ electrical depolarization throughout the cardiac cycle. The ECG results were assessed by a group of certified ECG medical practitioners. For each individual’s ECG, the certified staffs were responsible for evaluating the examination to label any identifiable issues (multiple issues allowed for one ECG). The ECG issues include: sinus bradycardia (heart rate < 60 beats/min), sinus tachycardia (heart rate > 100 beats/min), sinus arrhythmia (irregular timing between successive P waves), T wave change (T wave deviates from normal and appears as flat, oddly-shaped, or inverted), high voltage of the left ventricle (RV5 + SV1 > 4.0 mV for males and > 3.5 mV for females) etc. After ECG evaluation was completed for all cohort members, each individual was categorized into a binary phenotype of normal vs. abnormal ECG based on the presence or absence of labelled ECG issues. The “normal” refers to the group with none of the labelled issues (issue count = 0), and the “abnormal” covers individuals who display at least one out of the various subtypes of ECG issues (issue count ≥ 1). It should be noted that as the phenotype-of-interest, abnormal ECG in this study refers to the feature assessment regarding general ECG issues but does not directly infer clinical abnormity (as certain ECG issues could be non-specific and have no clinical implications).

Related health and personal information of the participants were collected through physical examination and face-to-face survey by trained staffs. For biological samples, participants spitted 1–2 ml of saliva into sterilized sample tubes, which were transported back to a genetic laboratory within 24 h and subjected to DNA purification. Upon quality validation, DNA samples were genotyped on a high-throughput Affymetrix Axiom Precision Medicine Array chip on a GeneTitan Multi-Channel Instrument platform (Thermo Fisher Scientific). The methods were carried out in accordance with the relevant guidelines and regulations.

### Data analysis

A dataset of cohort individuals with their categorized ECG status, genotypes, and related personal information (sex, age etc.) was assembled by the completion of on-site ECG measurements and lab experiments. Only SNPs on autosomes were selected. Genetic dataset is pre-processed for quality checks at both individual and SNP levels. Identity-by-descent (IBD) analysis iteratively disqualifies individuals exceeding a kinship threshold to remove cryptic relatedness. Hardy–Weinberg equilibrium (HWE) test excludes potential population substructure or genotyping errors. Details of the data pre-processing steps were annotated (Table S1). Principal component analysis (PCA) on SNPs in linkage equilibrium leads to principal components (PCs) essential for the adjustment of remaining population substructure (the top ten PCs were included in statistical models as covariates for adjustment). For GWAS, the model of multivariate logistic regression was fitted with the binary ECG status phenotype as the dependent variable, and the genotype and covariates (sex, age, PCs) as the independent variables. Genotypes were coded as the copies of minor allele, and accordingly the additive effect model was selected. A stringent Bonferroni-corrected threshold of 2 × 10^–7^ (significance level of 0.05 adjusted for about 250,000 SNPs; 243,487 SNPs analyzed for GWAS) and a less stringent candidate threshold of 2 × 10^–5^ were used exclusively for screening hit and candidate SNPs. Statistical analysis and data visualization were conducted in version 4.0.2 of the R statistical environment (R core team, 2020); parallel computing in GWAS was run on a Windows computer with an Intel CPU (32G RAM, 3.6 GHz, 8 cores).

### Ethics approval

All participants in this study have signed the Informed Consent Form before participation to allow their data for scientific research purposes only. The health and genetic data from measurements were subjected to strict anonymization for the respect and protection of personal privacy. In observance of the Regulatory Articles of Human Genetic Resources of P.R. China, all work of genotyping and data analysis were conducted in China, and individuals’ genetic data could not be made accessible without official authorization and approval from the supervisory agencies. The study design and procedure were approved by the Research Ethics Committee at West China School of Public Health, Sichuan University.

## Results

**Figure 1:**
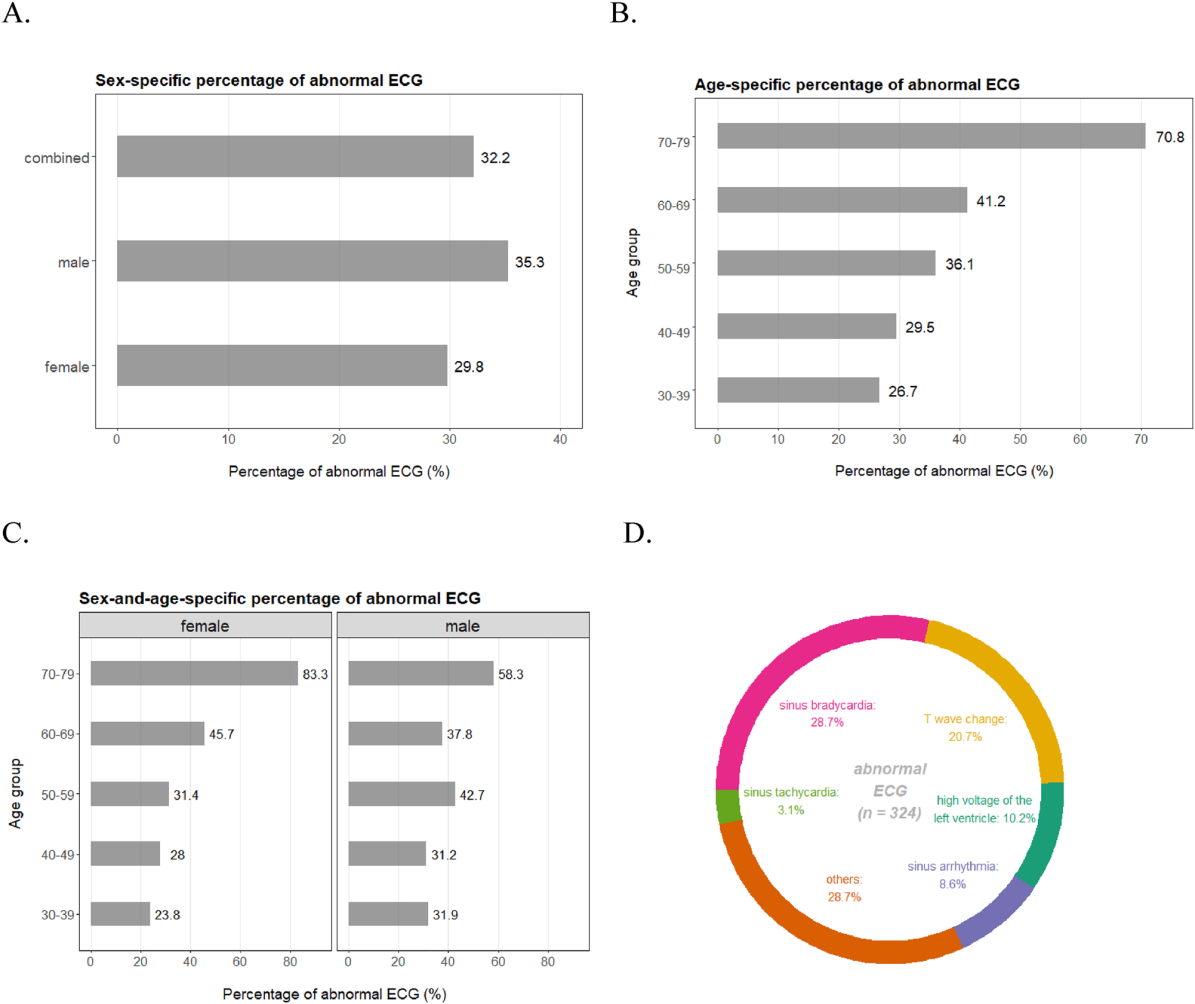
Percentage of abnormal ECG assessment by sex (**A**), age (**B**), sex and age (**C**), and subtypes of ECG issues (**D**) in the cohort.

With regard to the sex ratio, 439 (43.6%) of the participants are males while the other 567 (56.4%) are females so the cohort is slightly unbalanced towards the females (two-sample proportion test P = 6.2 × 10^–5^). The median age of the cohort is 46 years-old, and there is no apparent difference in age distribution regarding sex (Figure S1) or the ECG phenotype (Figure S2). For the outcome-of-interest of professional ECG assessment, 682 (67.8%) participants are evaluated as normal while the other 324 (32.2%) individuals are attributed to at least one ECG subtype issue so classified as abnormal. Percentages of abnormal ECG are 35.3% for the males and 29.8% for the females, with the difference being marginally non-significant (two-sample proportion test P = 0.07) (Fig. 1A). In comparison, there is apparently an upward trend in abnormal ECG with increasing ages (Cochran-Armitage trend test P = 7.8 × 10^–6^): notably, 70.8% of the most senior group (70–79 years-old) displays at least one subtype of issues in ECG assessment (Fig. 1B). Such observation is consistent with the general notion that as people age, their cardiac functions would gradually degenerate. The age-dependent sequential trends are well observed in the females but to a less extent in the males (Fig. 1C). It is also of interest that for abnormal ECG, females display lower percentage than males during middle-ages (30–59 years-old) but the contrast is reversed for senior ages (60–79 years-old). Nevertheless, sex and age are influential factors associated with the ECG results, and thus would be included as covariates for adjustment in the following analysis of GWAS. Among the detailed composition of ECG issues, the top three types combined account for around 60% of all cases and are respectively sinus bradycardia (28.7%), T wave change (20.7%), and high-voltage of the left ventricle (10.2%) (Fig. 1D).

A number of previous GWASs aimed to investigate the genetic association underlying electrocardiography, and their phenotypes-of-interest are specific quantitative metrics of the ECG such as QT interval, PR interval, QRS-T angle etc. A collection of 27 highly significant associations have been annotated with electrocardiography in the GWAS Catalog database^41^ (Table S2). 11 out of the curated SNPs are available on our gene chip, and none of them display any apparent association with ECG abnormity (Table S3). A major reason for this disparity may lie in the different types of measurements for the phenotype: a qualitative normal vs*.* abnormal ECG assessment in this study compared to quantitative features of ECG in all previous studies.

**Figure 2:**
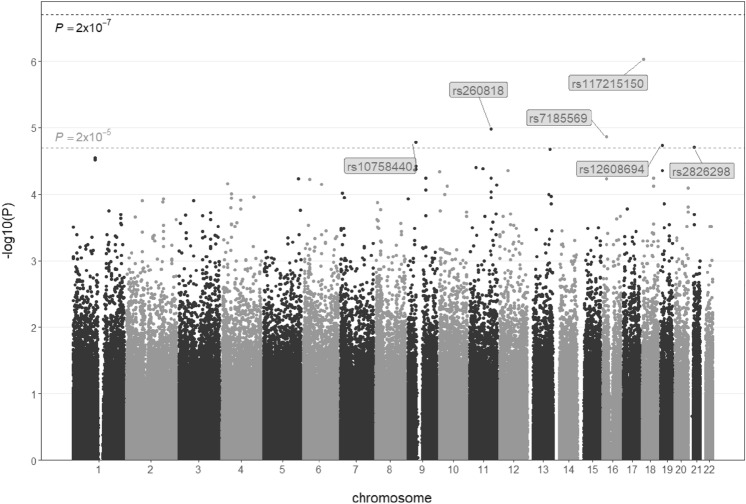
Manhattan plot of GWAS on the phenotype of normal vs. abnormal ECG. Bonferroni corrected threshold (2 × 10^–7^) and less stringent candidate threshold (2 × 10^–5^) respectively correspond to levels of 6.7 and 4.7 for − log_10_(P).

All participants in this study are Chinese of Han ethnicity, and principal component analysis validates the relative homogeneity of the cohort. A GWAS is conducted to map the genetic components linked to the observed abnormal ECG in this cohort. Due to the nature of ECG abnormity as a complex trait and as well the limited sample size in such pilot trial, both a Bonferroni-corrected level of 2 × 10^–7^ and a 100-times less stringent candidate level of 2 × 10^–5^ are defined as thresholds of significant and candidate associations. No variant scores high enough above the Bonferroni-corrected level, but a list of six SNPs surpass the candidate level (Fig. 2; Table 1). The candidate SNPs locate to different chromosomes without leading to any changes to the primary sequence of a gene product. In Hardy–Weinberg equilibrium, four of the six SNPs confer higher risk and the other two contribute to lower risk of abnormal ECG, and the allele frequency is comparable among different ethnicities across the 1000 Genomes^42^ (Figure S4). The candidate SNPs display significant but relatively weak association with abnormal ECG as revealed by the respective parameters of classification performance (Table 2). For the covariates in GWAS, age is significant with positive coefficients, and the females have higher risk compared to the males (Table S4), both in agreement with the above descriptive analysis of the cohort.

**Table 1:** Summary of candidate SNPs from GWAS.

SNP	Chr. (position)	Gene	Ref/minor allele (%)	HWE	Coefficient	P value	OR	95% CI_OR_
*rs117215150*	18 (6717603)	Intergenic*	T/C (8.3%)	0.85	0.868	9.3 × 10^–7^	2.38	[1.68, 3.37]
*rs260818*	11 (103920105)	PDGFD (intron)	A/C (40.7%)	0.21	− 0.486	1.0 × 10^–5^	0.61	[0.50, 0.76]
*rs7185569*	16 (19446926)	TMC5 (intron)	T/C (13.8%)	0.41	0.612	1.3 × 10^–5^	1.84	[1.40, 2.43]
*rs10758440*	18 (37573330)	FBXO10 (intron)	A/C (12.9%)	0.91	0.645	1.6 × 10^–5^	1.91	[1.42, 2.56]
*rs12608694*	19 (9361302)	OR7E24 (upstream)	T/C (38.0%)	0.13	− 0.464	1.8 × 10^–5^	0.63	[0.51, 0.78]
*rs2826298*	21 (21848839)	Intergenic*	C/T (7.1%)	0.55	0.813	2.0 × 10^–5^	2.25	[1.55, 3.27]

*Chr*. Chromosome, *position* GRCh37 coordinate, *HWE* hypothesis test for Hardy–Weinberg equilibrium, *OR* odds ratio.

*rs117215150 lies between LOC100130480 and ARHGAP28; *rs2826298* lies between TMPRSS15 and NCAM2.

**Table 2:** Classification performance by candidate SNPs.

SNP	Accuracy	Sensitivity	Specificity	PPV	NPV	F1	AUC	Youden
*rs117215150*	0.635	0.505	0.696	0.439	0.749	0.470	0.632	0.209
*rs260818*	0.594	0.599	0.591	0.408	0.758	0.486	0.626	0.210
*rs7185569*	0.625	0.556	0.658	0.432	0.760	0.487	0.632	0.232
*rs10758440*	0.611	0.541	0.645	0.421	0.747	0.473	0.626	0.206
*rs12608694*	0.595	0.590	0.598	0.408	0.757	0.483	0.633	0.210
*rs2826298*	0.604	0.497	0.654	0.402	0.736	0.444	0.617	0.187

Parameters of classification performance were calculated using the percentage of abnormal ECG in the cohort as the threshold.

*PPV/NPV* positive/negative predictive value, *AUC* area under the ROC curve, *Youden* Youden index.

**Figure 3:**
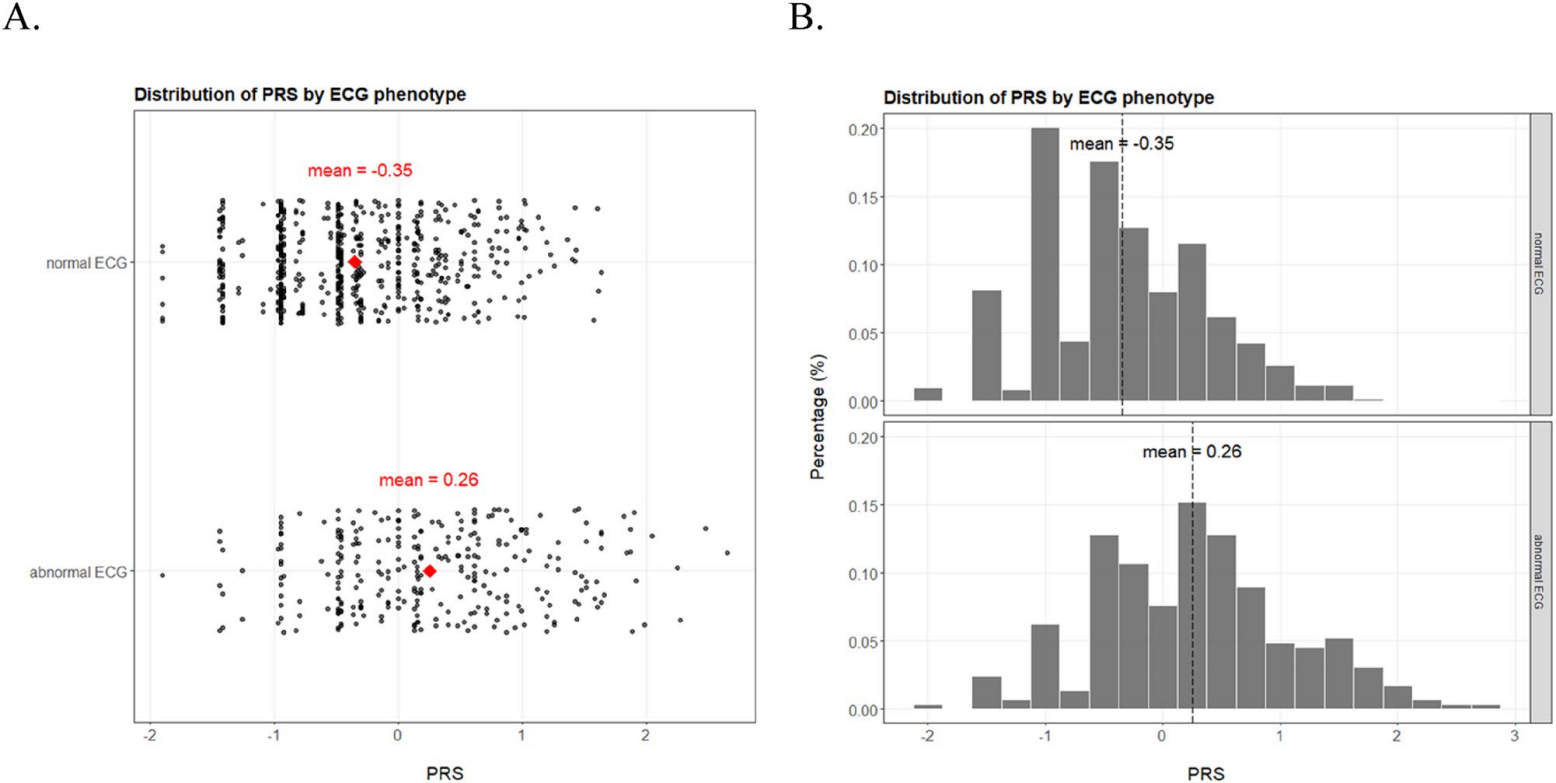
Distribution of PRS by ECG phenotype in the jittered dot plot (**A**) and the histogram (**B**).

**Figure 4:**
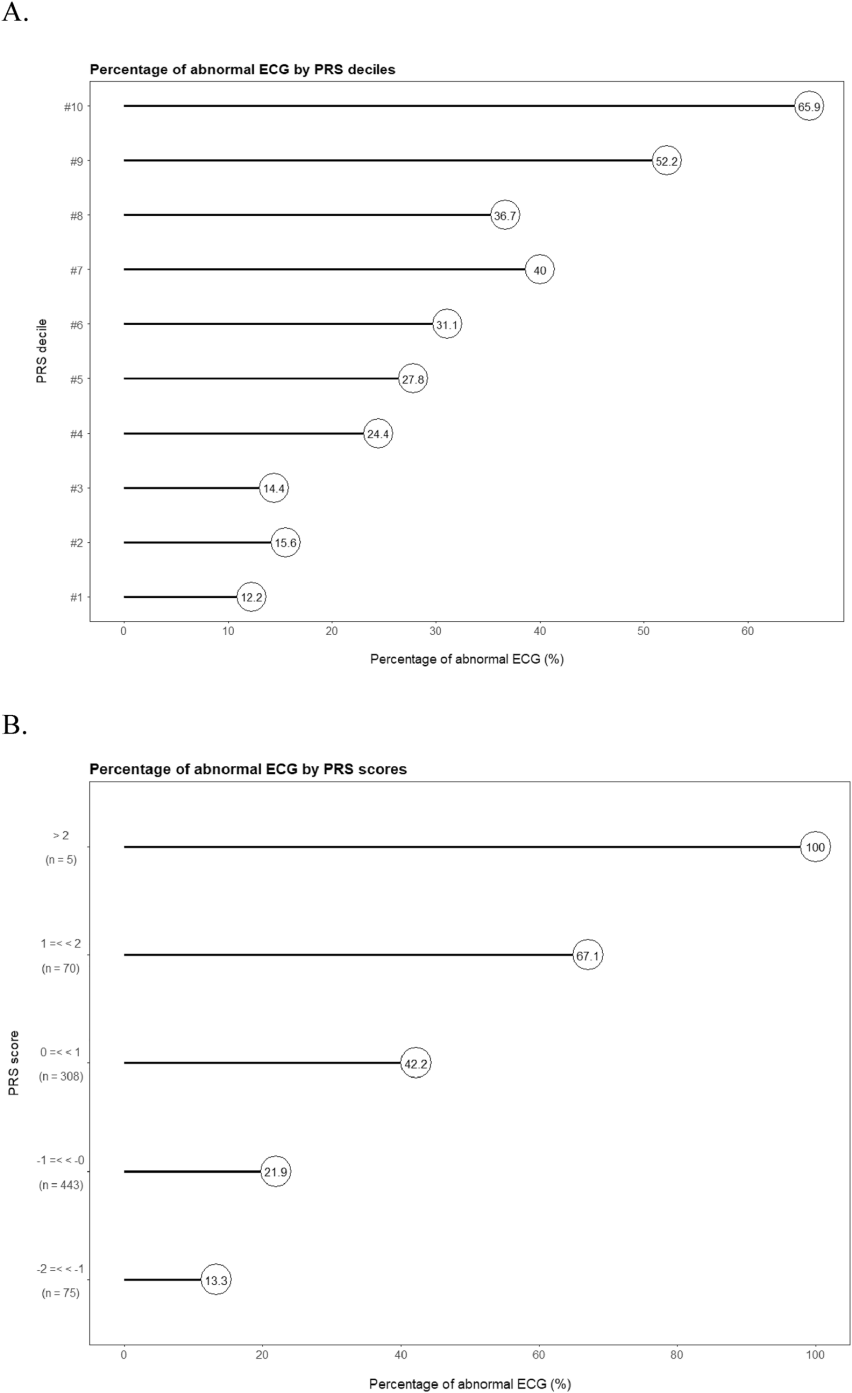
Percentage of abnormal ECG in subgroups of PRS deciles (**A**) and PRS scores (**B**). The ten deciles refer to the sequential 10% segments of the cohort ordered by the PRS values. For example, decile #1 annotates individuals with the lowest 10% PRS, and decile #10 annotates individuals with the highest 10% PRS.

With ECG assessment being a complex trait and thus a non-Mendelian phenotype, single SNPs would not, in agreement with our GWAS findings, confer strong predictive power. Instead, combination of the candidate SNPs would result in more informative and robust features, such as polygenic risk scores. We calculate a polygenic risk score (PRS) with the six candidate SNPs weighted by their respective coefficients (Figure S5). The PRS is significantly different in the case–control perspective between normal ECG and abnormal ECG (Wilcoxon rank sum test P = 2 × 10^–16^, Fig. 3). More convincingly, the PRS displays strong association with abnormal ECG as increasing PRS correlates well with the susceptibility to abnormal ECG (Fig. 4). The highest and lowest deciles of PRS display proportion of abnormal ECG at 65.9% and 12.2% respectively, well differentiating the cohort (abnormal ECG at 32.3%) into the high vs*.* low risk segments (Fig. 4A). The PRS values also match the observed pattern, and the risk is guaranteed for the few individuals with PRS over 2 (Fig. 4B).

**Figure 5:**
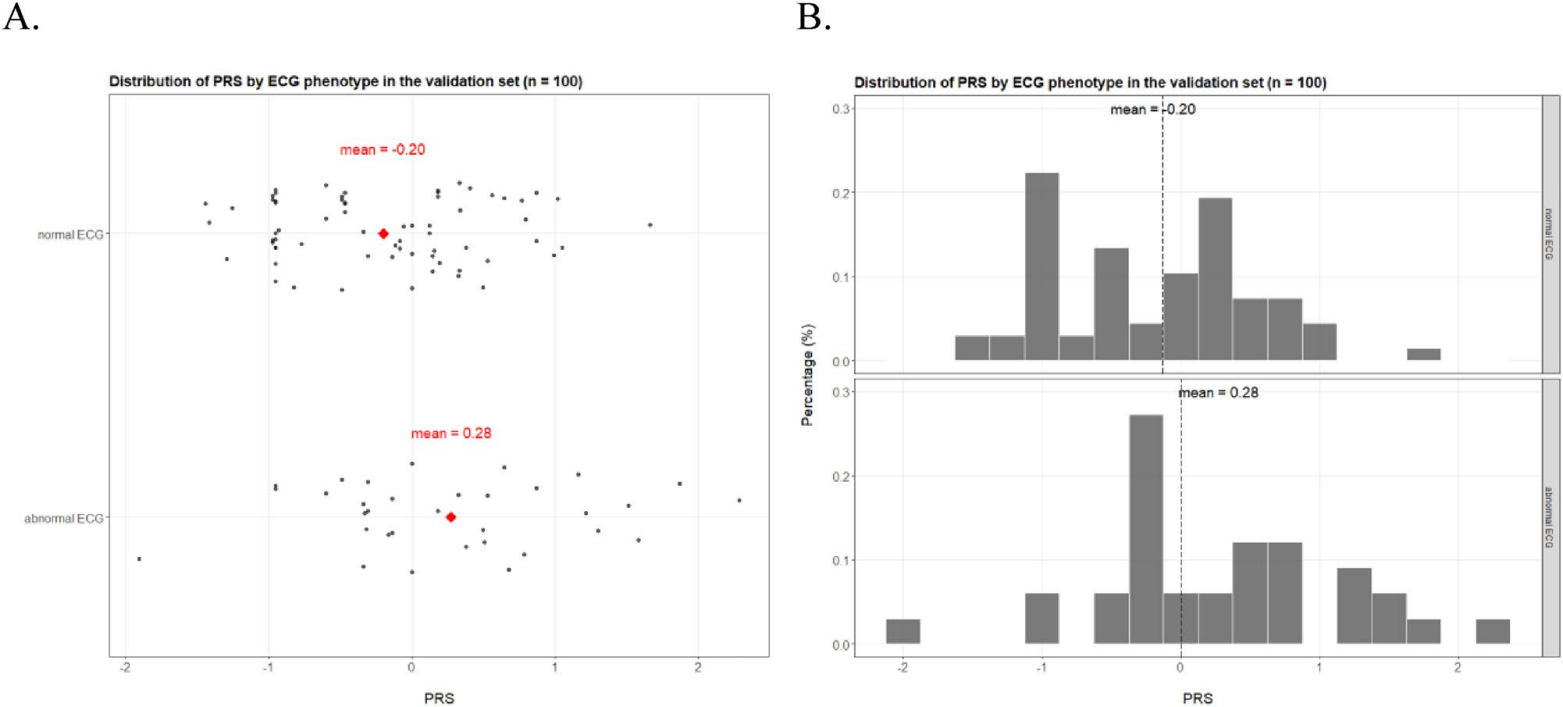
Distribution of PRS by ECG phenotype in the jittered dot plot (**A**) and the histogram (**B**) for the validation set.

**Figure 6:**
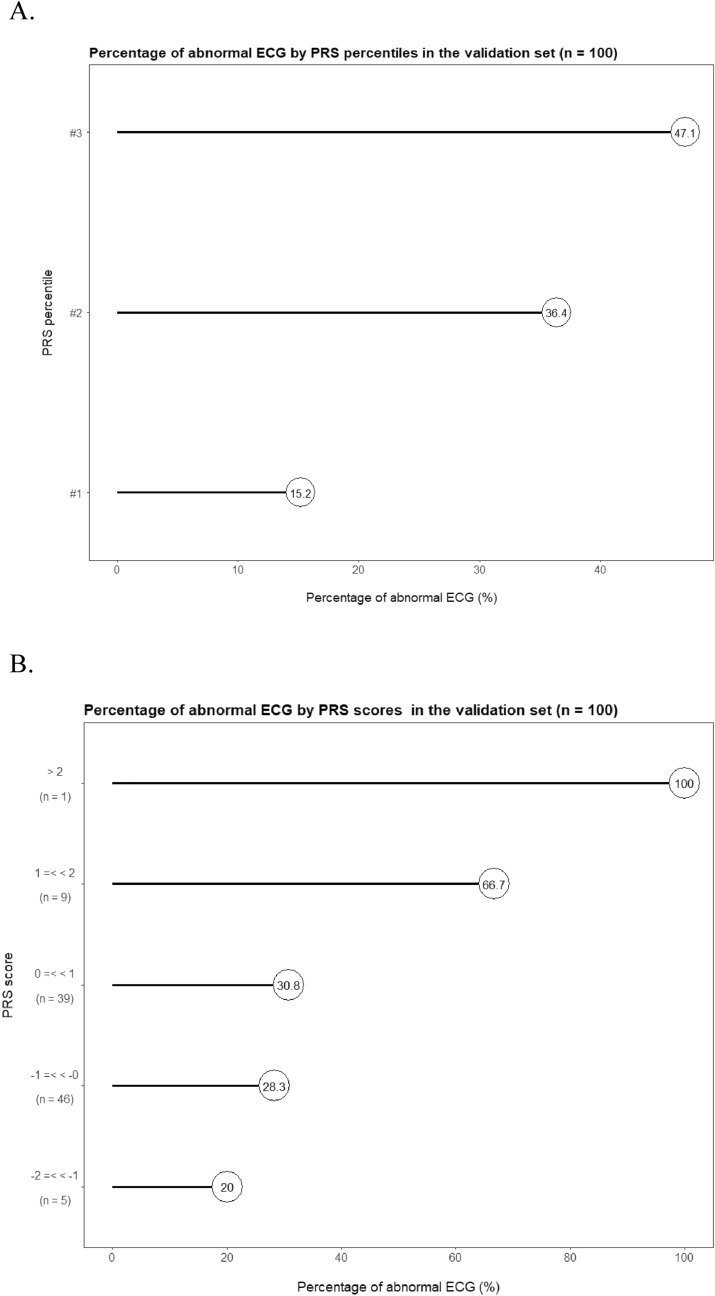
Percentage of abnormal ECG in subgroups of PRS percentiles (**A**) and PRS scores (**B**). Percentile #1, #2, and #3 respectively annotates the low, middle, and high one-third of PRS individuals. The validation set is divided into three percentiles rather than ten deciles due to its limited sample size.

**Figure 7:**
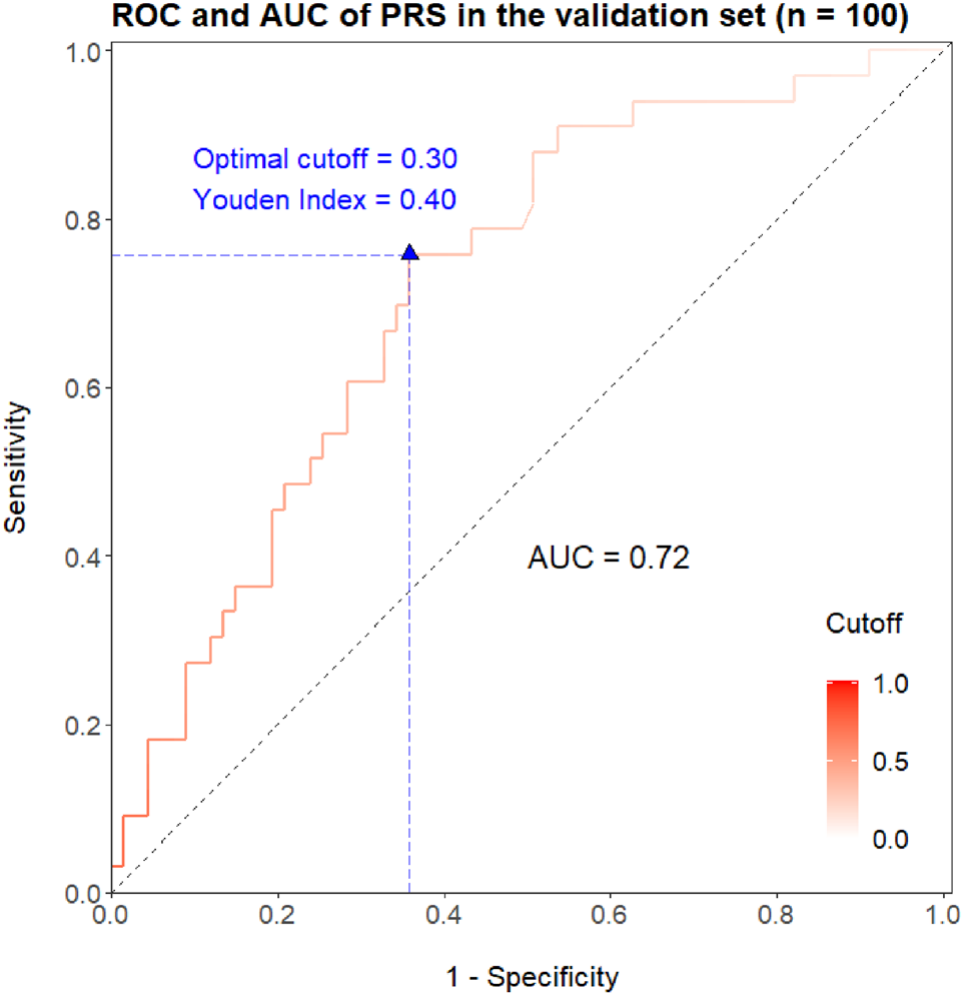
ROC plot of the PRS in the validation set. Cutoff (in the range of [0, 1]) refers to the threshold to dichotomize predicted probability into binary classes. *AUC* area under the ROC curve. Note: sex and age are included in the predictive model.

The GWAS and PRS evaluations above are carried out in the same cohort, so there would be potential issues of overfitting. To address such concerns, we recruit an independent cohort of 100 individuals as the validation set to objectively assess the robustness and effectiveness of the candidate SNPs and the PRS. The limited sample-size of the validation set leads to the rarity of homogenous minor allele individuals, and consequently, associations of the candidate SNPs to abnormal ECG could not be effectively confirmed in the perspective of hypothesis test (P value under 0.05 for only one SNP) (Table S5). However, all six candidate SNPs display, in the validation set, classification performances that are better than random guess and similar to those in the initial discovery cohort (Table S5), thus confirming the presence of candidate associations in an independent population. In the validation set, the PRS displays the similar unimodal distribution (Figure S6), is different between the normal and abnormal groups (Wilcoxon rank sum test P = 0.01, Fig. 5), and reproducibly correlates well in a unidirectional trend with the susceptibility of abnormal ECG (Fig. 6). The AUC reaches 0.72 and the Youden index is at 0.40 in the ROC curve for the validation set (Fig. 7), further substantiating the PRS as a potential biomarker for high-risk subgroup of abnormal ECG in the Chinese population.

## Discussion

A clear uptrend of abnormal ECG with regard to aging is observed in the cohort, matching the progressive deterioration of cardiovascular functions throughout individuals’ lifetime. On the other hand, sex also plays a role as the males and the females display similar but not identical pattern regarding the percentage of abnormal ECG. Therefore, incorporating age and sex into a statistical learning model is instrumental to facilitate the robust forecast of individuals’ risk in being assessed for abnormal ECG.

While previous GWASs on electrocardiography were mainly focused on the specific quantitative metrics of ECG, one unique feature of this study is the categorization of ECG assessments into a general normal *vs.* abnormal binary phenotype based on the labelling of potential issues by certified medical staffs. This partially explains why previously identified genetic variants do not score significantly in this study, similar to the phenomenon observed in the treatment of blood pressure as a quantitative trait (e.g. systolic pressure) *vs.* as a qualitative trait (e.g. hypertension)^43,44^. In addition, different backgrounds of ethnicity in these studies might also contribute to the disparate hits of genetic variants. Undoubtedly, there are various subtypes of ECG issues so generalization of all issues into a single abnormal group may be possibly confounding. However, participants may display multiple subtypes (some individuals in this study were labelled for up to 5 ECG issues) so the subtypes are themselves complex and non-independent outcomes to evaluate for individuals. Moreover, both the examination and the evaluation of ECG and as well the genotyping of a cohort are time and resource consuming, so subtype-based analysis suffers from significantly reduced sample size which is the major obstacle for discovering positive hits in heavily-adjusted genomic analysis. By evaluating ECG assessments into a binary outcome, this study benefits from the statistical power conferred by the full cohort. Subtype association analysis would be better suited if sample size would expand for our cohort in the future or when established large-scale cohorts such as the UK Biobank are analyzed^45^.

Abnormal ECG proves to be a complex trait for the mapping to hit genetic variants. None of the six candidate SNPs identified in this study lead to alterations in the primary sequence of proteins, but four of them locate to the noncoding regions of certain genes: *PDGFD* (platelet derived growth factor D), *TMC5* (transmembrane channel like 5), *FBXO10* (F-box protein 10), and *OR7E24* (olfactory receptor family 7 subfamily E member 24). The mechanisms underlying these potential associations remain elusive but factors such as transcriptional regulation or alternative splicing might be involved. Higher statistical power from a larger-size cohort would better reveal the genetic-phenotypic link. Nevertheless, assembling information from all six candidate SNPs, a polygenic risk score displays robust and reproducible difference between the normal and abnormal groups, and sequentially correlates with the increased risk of abnormity in ECG. Validation of the PRS features in the independent set not only supports the credibility of our GWAS but also suggests the potential application of the PRS as a novel biomarker to early detecting Han Chinese individuals with high-risk of heart issues in a precision medicine perspective.

To our knowledge, this is the first GWAS using the professional assessments of ECG as the phenotype-of-interest, and it remains to be investigated to what extent our discoveries in a Han Chinese cohort could be reproduced in other ethnicities. It should also be noted that neither the SNPs nor the PRS lead to optimal classification performance, suggesting the genetic association only contributes partially to the phenotype of abnormal ECG. This should not be surprising because ECG status is itself a complex trait that should be influenced and determined by many factors (genetic, environmental, life-style etc.). Future research should aim to collect information on such variables within large cohorts in a longitudinal timeframe to enhance both the association analysis and the predictive modeling of ECG features and status.

## Supplementary Information

Supplementary Information.

## Data Availability

The Informed Consent Form for the cohort study states that only population-based results would be published and any individual’s raw genetic data are prohibited from unauthorized release. Consequently, cohort summary results are reported for GWAS in the perspective of population genetics. Summary statistics for the set of SNPs can be individually inquired from a publicly accessible user-interactive website (https://mengqiao-wang.shinyapps.io/GWAS_ECG).
