# Author Correction: A genome-wide association and polygenic risk score study on abnormal electrocardiogram in a Chinese population

**DOI:** 10.1038/s41598-021-01355-7

**Published:** 2021-11-01

**Authors:** Mengqiao Wang, Jiaqi Gao, Yang Shi, Xing Zhao

**Affiliations:** 1 Department of Epidemiology and Biostatistics, West China School of Public Health and West China Fourth Hospital, Sichuan University, Renmin South Road 16, Chengdu, 610041 Sichuan People’s Republic of China; 2 Department of Population Health Science, Medical College of Georgia, Augusta University, 1120 15th Street, Augusta, GA 30912 USA

Correction to: *Scientific Reports* https://doi.org/10.1038/s41598-021-84135-7, published online 25 February 2021

The Funding section in the original version of this Article was incomplete.

“This work was supported by the National Key Research and Development Program of China (2017YFC0907305 and 2017YFC0907300) and the Basic Research Grant from the Department of Science and Technology of Sichuan Province (2019YJ0035).”

now reads:

“This work was supported by the National Key Research and Development Program of China (2017YFC0907305 and 2017YFC0907300), the Youth Scholar Grant from the National Natural Science Foundation of China (81800071), and the Basic Research Grant from the Department of Science and Technology of Sichuan Province (2019YJ0035).”

The original Article has been corrected.

