# Supplementary material for: A genome-wide association and polygenic risk score study on abnormal electrocardiogram in a Chinese population
Source: Sci Rep. 2021 Feb 25;11:4669. doi: 10.1038/s41598-021-84135-7 (PMC7907205; doi:10.1038/s41598-021-84135-7)
Supplement: Supplementary file 1 — Supplementary Information. [file 41598_2021_84135_MOESM1_ESM.docx]

**Supplementary material**

A genome-wide association and polygenic risk score study

on abnormal electrocardiogram in a Chinese population

Mengqiao Wang^1^*, Jiaqi Gao^1^, Yang Shi^2^*, Xing Zhao^1^*

^1^ Department of Epidemiology and Biostatistics, West China School of Public Health and West China Fourth Hospital, Sichuan University, Renmin South Road 16, Chengdu, Sichuan Province, 610041, P.R. China

^2^ Department of Population Health Science, Medical College of Georgia, Augusta University, 1120 15th Street, Augusta, GA 30912, U.S.A

* corresponding author ([mengqiaowang@gmail.com](mailto:mengqiaowang@gmail.com);

[yshi@augusta.edu](mailto:yshi@augusta.edu);

[zhaoxing731@gmail.com](mailto:zhaoxing731@gmail.com))

**Supplementary figures**

**Figure S1**


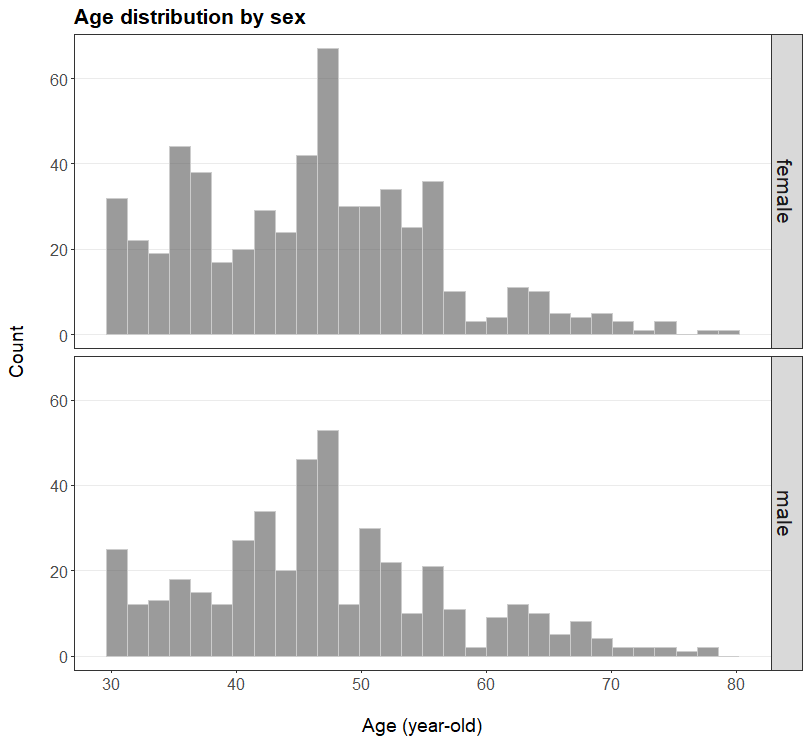


**Figure S2**


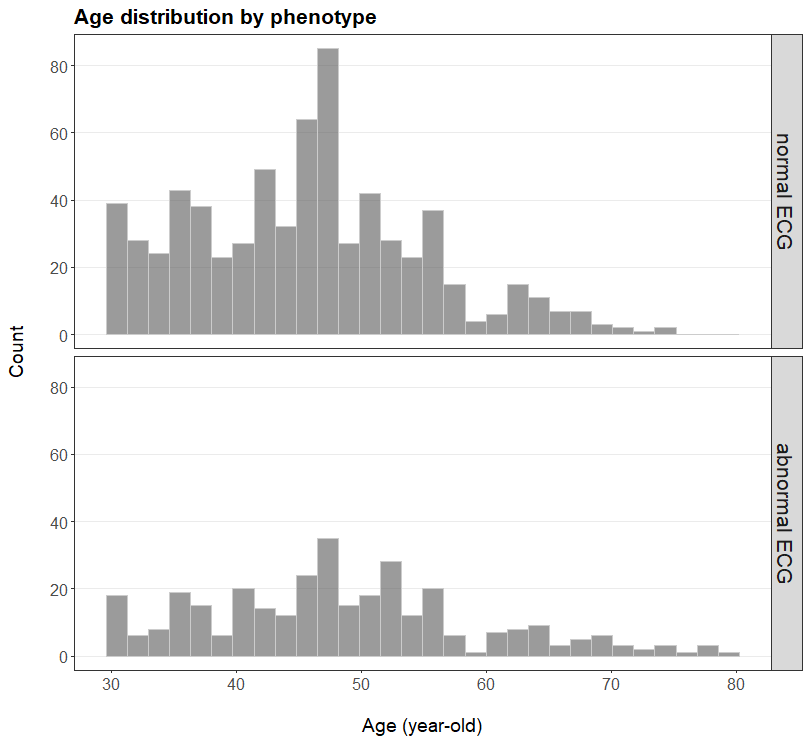


**Figure S3**


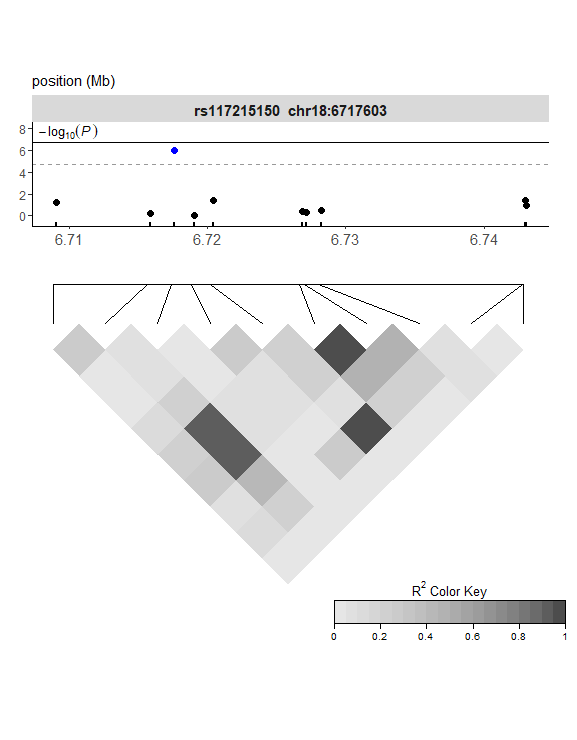

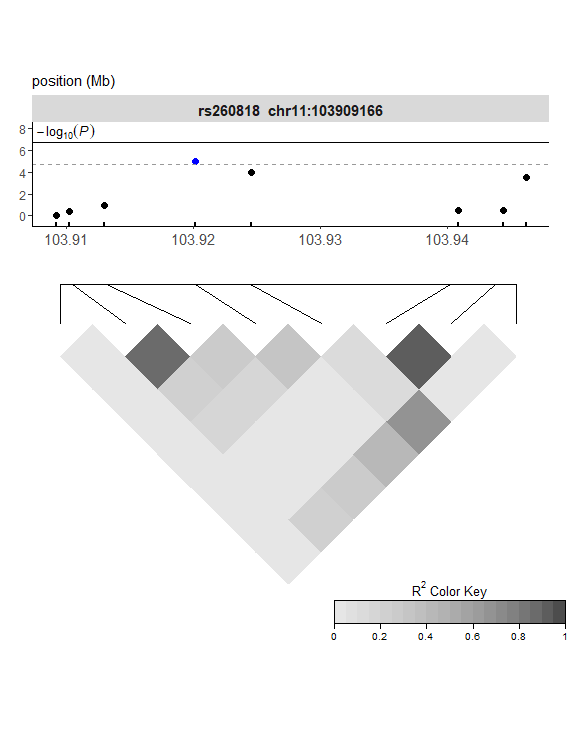

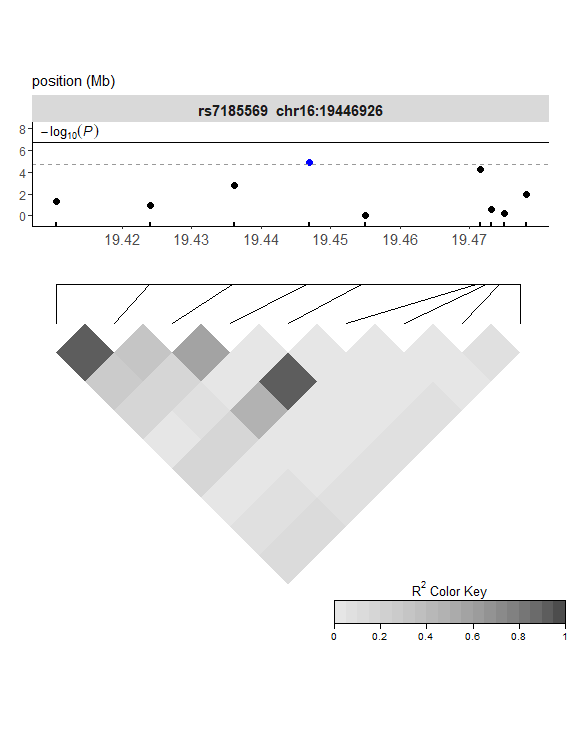


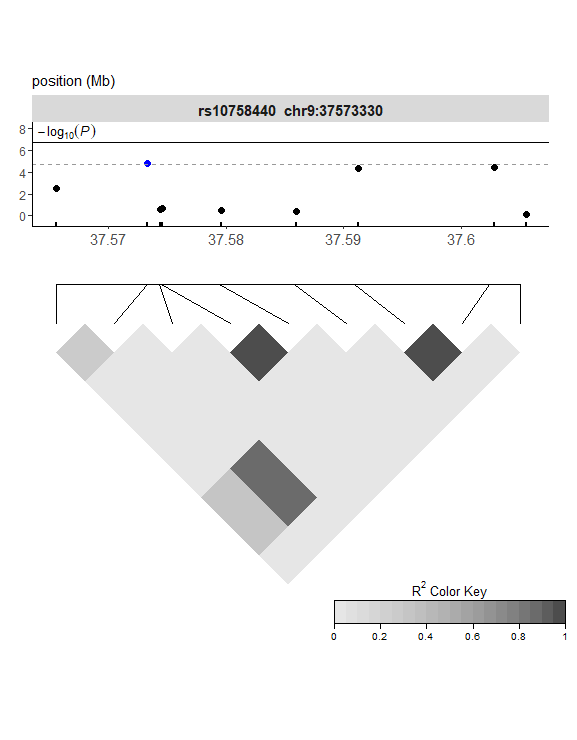

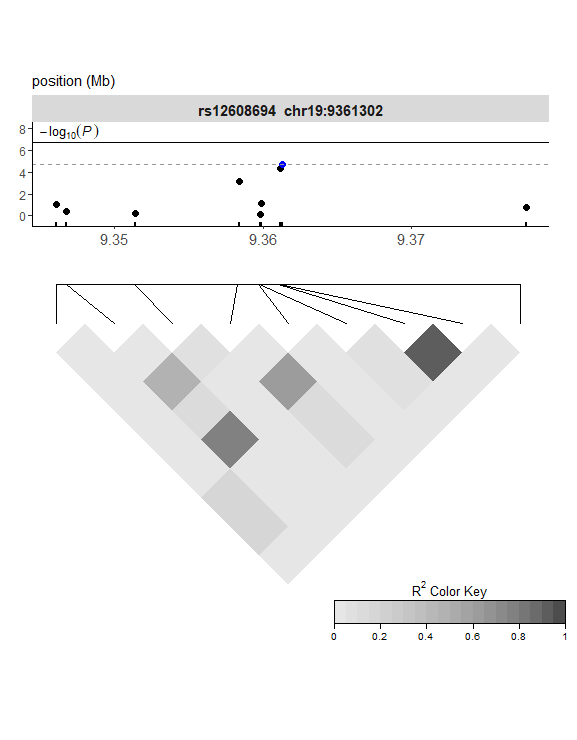

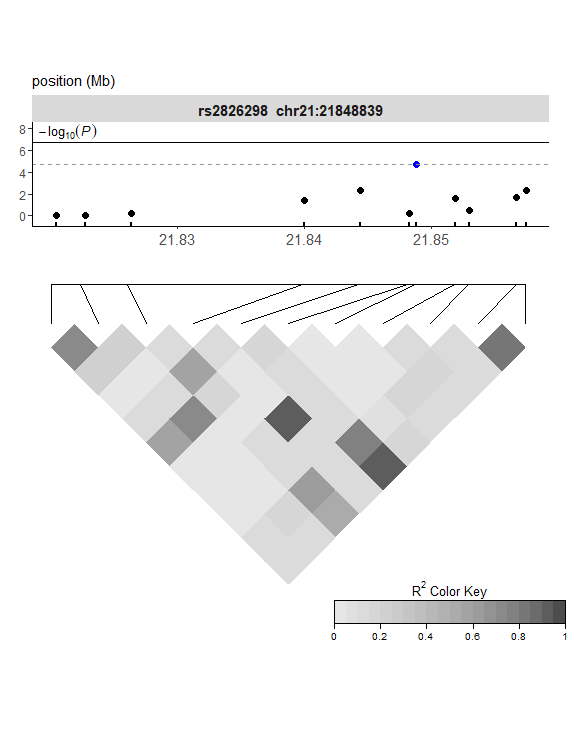


**Figure S4**


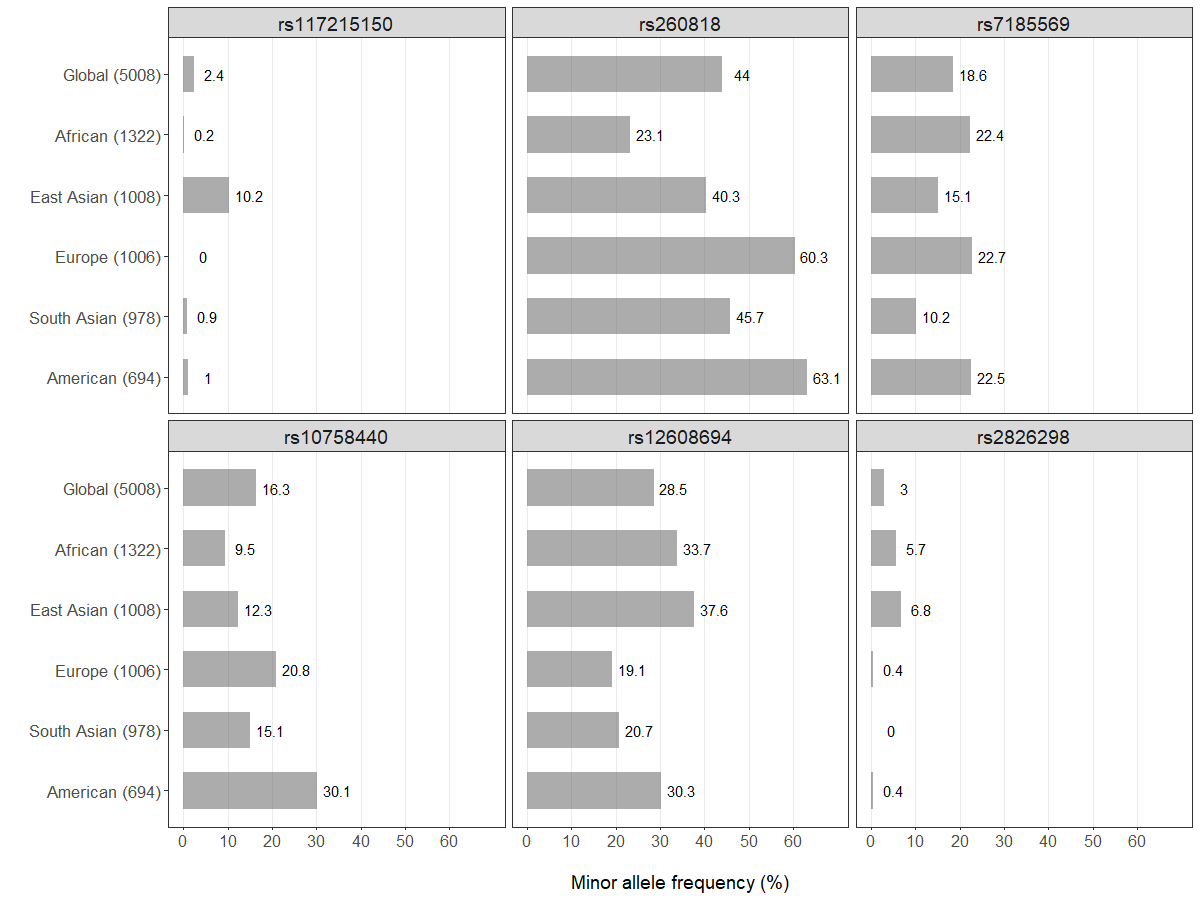


**Figure S5**


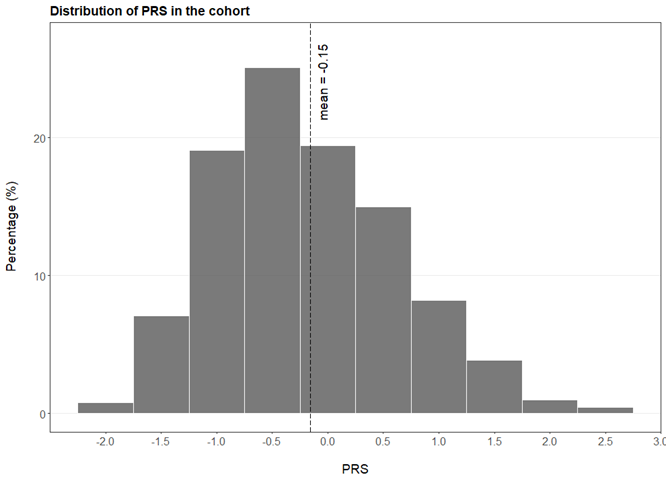


**Figure S6**


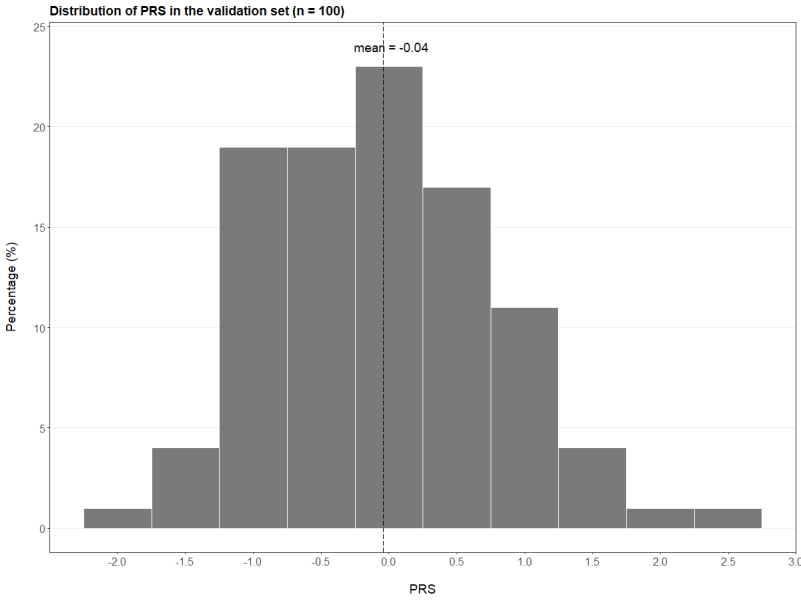


**Supplementary figure legends**

Figure S1. Age distribution by sex of the cohort.

Figure S2. Age distribution by ECG phenotype of the cohort.

Figure S3. Regional association plot of the candidate SNPs. Solid and dashed horizontal lines respectively annotate the Bonferroni-corrected hit and less stringent candidate thresholds.

Figure S4. Minor allele frequency of the candidate SNPs in the global and five super-ancestry groups from the 1000 Genomes. Numbers in the parenthesis annotate the respective sample sizes.

Figure S5. Distribution of PRS in the cohort.

Figure S6. Distribution of PRS in the validation set.

**Supplementary tables**

**Table S1. Pre-processing steps for GWAS on abnormal ECG**

| **Step** | **Pre-processing** | **Sample** | **SNP** | **Note** |
| --- | --- | --- | --- | --- |
| 1 | measurement of cohort’s ECG and assessment of ECG features | 1006 | / | evaluation and labelling of each ECG with any identifiable issues by certified medical staffs; |
| 2 | classification of cohort’s ECG status as a binary phenotype | 1006 | / | an individual with at least one identified ECG issue would be categorized into the “abnormal ECG” group; an individual whose ECG has no issues labelled would be categorized into the “normal ECG” group; |
| 3 | genotyping | 999 | 511883 | exclusion of 7 individuals who did not pass the quality-control of high-throughput genotyping; |
| 4 | SNPs on autosomes | 999 | 511883 | exclusion of 45151 non-autosome SNPs; |
| 5 | SNP-level filtering | 999 | 466732 | exclusion of 222961 SNPs due to low call rate (39886, threshold = 0.95) or low minor allele frequency (147086, threshold = 0.05) or both (35989); |
| 6 | sample-level filtering | 998 | 243771 | exclusion of 1 individual due to low call rate (1, threshold = 0.95) or high inbreeding coefficient (0, threshold = 0.1) or both (0); |
| 7 | LD pruning | 998 | 243771 | selection of SNPs with LD pruning (threshold = 0.2, sliding maximum window size of 500000 bps); |
| 8 | kinship filtering | 919 | 243771 | iterative exclusion of 79 individuals due to cryptic relatedness using identity-by-descent (IBD) kinship analysis (threshold = 0.1); |
| 9 | population substructure filtering | 919 | 243771 | check individuals for non-Han ethnicity or for outliers in the principle component analysis (PCA): none excluded; |
| 10 | HWE filtering | 919 | 243771 | exclusion of 284 SNPs due to the rejection of Hardy-Weinberg Equilibrium at cutoff of 1×10^-6^; |
| 11 | final cohort | 919 | 243487 | a final dataset prepared for GWAS. |

**Table S2. Summary of 27 associations with electrocardiography in GWAS Catalog**

|  | SNP | Location | P value | annotation | Beta | Mapped gene |
| --- | --- | --- | --- | --- | --- | --- |
| 1 | *rs10507380* | 13:27305389 | 8x10^-6^ | (QT interval) | NA | RNU6-70P |
| 2 | *rs882300* | 2:136218685 | 3x10^-7^ | (PR interval) | NA | AC112255.1,  HNRNPKP2 |
| 3 | *rs283566* | 6:51091008 | 9x10^-6^ | (P wave duration) | NA | FTH1P5,  AL158050.2 |
| 4 | *rs7638909* | 3:38553482 | 2x10^-6^ | (PR interval) | 4.8ms increase | SCN5A |
| 5 | *rs7512898* | 1:200703778 | 5x10^-6^ | (PR segment) | NA | CAMSAP2 |
| 6 | *rs2461751* | 2:175424591 | 8x10^-6^ | (PR interval) | 4.54ms increase | AC093459.1 |
| 7 | *rs2070488* | 3:38400999 | 4x10^-6^ | (PR interval) | 5ms decrease | XYLB |
| 8 | *rs2008242* | 4:5219811 | 3x10^-6^ | (PR segment) | NA | STK32B |
| 9 | *rs17030434* | 4:153782444 | 5x10^-6^ | (P wave duration) | NA | SFRP2 |
| 10 | *rs7301677* | 12:114943342 | 2x10^-24^ | (QRS-T angle) | 0.14unit increase | AC008125.1,  AC009804.1 |
| 11 | *rs7966951* | 12:114927406 | 2x10^-16^ | (SVG elevation) | 0.11unit increase | AC009804.1,  AC009804.2 |
| 12 | *rs1124477* | 12:114911692 | 1x10^-14^ | (SVG magnitude) | 0.1unit decrease | AC009804.1,  AC009804.2 |
| 13 | *rs10076436* | 5:154492281 | 2x10^-20^ | (SVG azimuth) | 0.12unit increase | AC026688.2,  AC026688.1 |
| 14 | *rs13165478* | 5:154489480 | 9x10^-9^ | (QRS-T angle) | 0.07unit decrease | AC026688.2,  AC026688.1 |
| 15 | *rs2207793* | 1:61428981 | 4x10^-11^ | (QRS-T angle) | 0.08unit decrease | NFIA |
| 16 | *rs2207793* | 1:61428981 | 2x10^-10^ | (SVG azimuth) | 0.08unit decrease | NFIA |
| 17 | *rs7029396* | 9:130263971 | 2x10^-10^ | (SAI QRST) | 0.09unit decrease | NCS1, HMCN2 |
| 18 | *rs7029396* | 9:130263971 | 2x10^-9^ | (SVG magnitude) | 0.09unit decrease | NCS1, HMCN2 |
| 19 | *rs4966020* | 15:98741451 | 1x10^-12^ | (SAI QRST) | 0.08unit increase | IGF1R |
| 20 | *rs7638275* | 3:38624332 | 3x10^-11^ | (SAI QRST) | 0.3unit decrease | SCN5A |
| 21 | *rs10799792* | 1:23119948 | 3x10^-9^ | (SAI QRST) | 0.08unit increase | LUZP1 |
| 22 | *rs2042400* | 16:58430436 | 4x10^-9^ | (SAI QRST) | 0.07unit decrease | LINC02137 |
| 23 | *rs11039216* | 11:47385041 | 6x10^-9^ | (SAI QRST) | 0.07unit increase | AC090559.1 |
| 24 | *rs2098226* | 7:5543414 | 8x10^-9^ | (SAI QRST) | 0.07unit increase | ACTB |
| 25 | *rs6795970* | 3:38725184 | 3x10^-15^ | (PR interval) | 3.33ms increase | SCN10A |
| 26 | *rs1415259* | 1:162115519 | 7x10^-10^ | (QTc interval) | 2.53ms decrease | NOS1AP |
| 27 | *rs4725982* | 7:150940775 | 3x10^-6^ | (QTc interval) | 2.08ms increase | AOC1, KCNH2 |

Note: last accessed on April 4, 2020 from GWAS Catalog (<https://www.ebi.ac.uk/gwas/efotraits/EFO_0004327>).

| **Table S3. GWAS Catalog associations with electrocardiography in this study**  SNP P value -log_10_(P)  1 *rs6795970* 0.333 0.480  2 *rs2098226* 0.428 0.370  3 *rs283566* 0.470 0.330  4 *rs4725982* 0.493 0.310  5 *rs882300* 0.497 0.300  6 *rs2461751* 0.589 0.230  7 *rs17030434* 0.732 0.140  8 *rs1415259* 0.812 0.090  9 *rs2070488* 0.830 0.080  10 *rs7512898* 0.855 0.070  11 *rs2008242* 0.970 0.010 |
| --- |

**Table S4. Summary of covariates from GWAS**

sex* age

SNP coefficient P value coefficient P value

*rs117215150* 0.239 0.105 0.024 0.001

*rs260818* 0.290 0.048 0.026 <0.001

*rs7185569* 0.259 0.077 0.024 0.001

*rs10758440* 0.306 0.038 0.024 0.001

*rs12608694* 0.276 0.060 0.026 <0.001

*rs2826298* 0.287 0.051 0.022 0.004

* with male as the reference level, the coefficient for sex is for female.

**Table S5. Summary of candidate SNPs in the validation set**

SNP AA Aa aa coefficient P value accuracy sensitivity specificity PPV NPV F1

*rs117215150* 79 20 1 0.385 0.429 0.600 0.576 0.612 0.422 0.745 0.487

*rs260818* 38 48 14 0.277 0.408 0.640 0.697 0.612 0.469 0.804 0.561

*rs7185569* 75 23 2 0.024 0.958 0.630 0.697 0.597 0.460 0.800 0.554

*rs10758440* 73 25 2 0.130 0.766 0.610 0.697 0.567 0.442 0.792 0.541

*rs12608694* 35 47 18 -0.448 0.171 0.640 0.636 0.642 0.467 0.782 0.538

*rs2826298* 82 15 3 1.364 0.007 0.670 0.545 0.731 0.500 0.766 0.522

A: major allele; a: minor allele.
